# A Qualitative Exploration of the Female Experience of Autism Spectrum Disorder (ASD)

**DOI:** 10.1007/s10803-019-03906-4

**Published:** 2019-02-21

**Authors:** Victoria Milner, Hollie McIntosh, Emma Colvert, Francesca Happé

**Affiliations:** Social, Genetic and Developmental Psychiatry Centre, Institute of Psychiatry, Psychology and Neuroscience, King’s College London, 16 De Crespigny Park, Denmark Hill, London, SE5 8AF UK

**Keywords:** Autism, Female autism, Sex differences, Experience, Gender

## Abstract

Autism spectrum disorder is thought to be a predominantly male diagnosis, however recent research suggests a smaller gender disparity in prevalence than previously assumed. Accounts of the female experience of autism are important to help reduce likely male-bias in current understanding and recognition of autism. Eighteen autistic females and four mothers of autistic females took part in discussions with a topic guide around diagnosis, impact and coping. A thematic analysis was conducted. Five themes were identified: fitting in the with the norm, potential obstacles for autistic women and girls, negative aspects of autism, the perspective of others, and positive aspects of having autism. We hope that greater understanding of the experiences of autistic females may lead to improved awareness, diagnosis and support for women and girls.

**Electronic supplementary material:**

The online version of this article (10.1007/s10803-019-03906-4) contains supplementary material, which is available to authorized users.

The current qualitative study explores female presentation and experience of autism spectrum disorder (ASD). ASD, hence forth referred to as “autism”, is a neurodevelopmental condition characterised by persistent difficulties in social interaction, social communication and restricted and repetitive patterns of behaviour, from a young age (Diagnostic and Statistical Manual of Mental Disorders-5th edition [*DSM–5*]; American Psychiatric Association (APA) 2013).

A striking feature of autism is the high male to female ratio, which has traditionally been reported to vary across the spectrum; most studies quote 4–5:1, falling to 2:1 where autism is accompanied by significant intellectual disability, and rising to perhaps 10:1 amongst autistic individuals with average or superior intellectual ability (Dworzynski et al. 2012). These ratios have been challenged more recently, however, by findings from epidemiological studies with active case ascertainment, which reveal significantly smaller male to female ratios in autism within general population groups (e.g., 2.5:1; Kim et al. 2011). A recent meta-analysis (Loomes et al. 2017) concluded that the ratio as estimated by methodologically rigorous studies, is likely to be 3:1, and may not change very much across the spectrum or intellectual ability range.

The lower male preponderance in epidemiological studies with active ascertainment, compared to those relying on clinical or educational records, suggests we are under-diagnosing autism in females. A number of reasons for this can be proposed. These include the use of solely male samples in some autism research, which has likely led to a biased understanding of the full spectrum of autism and its manifestations. Lai et al. (2015) noted that there is an ascertainment gender bias of up to 15:1 in neuroimaging research. Additionally, it has been suggested that recognition of autism and the current methods of diagnosis are based on stereotypes of autism as a male condition (Kopp and Gillberg 1992). Extrapolating a largely male model of autism to women and girls could be problematic if, as research suggests, the phenotypic presentation of autism often differs in women (Kirkovski et al. 2013). Lai et al. (2011) investigated behavioural difference in autistic males and females and found that females reported more lifetime sensory symptoms and fewer socio-communication difficulties than males. Furthermore, it has been suggested that compared to autistic males, autistic females are more able to demonstrate reciprocal conversation and are more motivated to initiate friendships (Lai et al. 2015). The “special interests” that autistic females adopt may also appear less unusual, focusing on topics similar to those of their neurotypical peers, such as an interest in celebrities or horses; however, the intensity and quality of the interests remain unusual (Gould and Ashton-Smith 2011). In addition, consciously copying neurotypical females and employing so-called ‘camouflaging’ may be common amongst autistic women and girls, perhaps contributing to under-diagnosis (Gould and Ashton-Smith 2011; Attwood 2006). All of these factors may play a part in exaggerating the male preponderance in autism, and result in autistic females not receiving much-needed diagnosis and support, with negative effects on their mental health and well-being (Pellicano et al. 2014).

Previous qualitative research into the female experience of autism confirms many of these issues. Bargiela et al. (2016) conducted a qualitative study with a group of adult autistic women (n = 14, aged 18–35 years) whose autistic tendencies had gone unrecognised up to their late teens. They describe the technique of “masking” as a common but not universal tool, used to disguise autistic traits in order to fit in. For several participants, it involved mimicking (an unconscious attempt) or learning (a conscious effort) socially acceptable behaviour. Generally, this was reported as being due to pressure to meet the expectations a neurotypical world imposes. While the women reported they were often successful at masking, it appeared to be a relatively superficial method of coping, with resultant difficulties ranging from constant exhaustion to one woman describing a loss of her own sense of identity (Bargiela et al. 2016). Furthermore, the use of camouflaging behaviours has been linked to increased self-reported stress and anxiety in comparison to those who do not camouflage (Cage and Troxell-Whitman 2019; Hull et al. 2017).

The desire to fit in with neurotypical peers may also influence the use of camouflaging behaviours. An interview study reported autistic girls (n = 10, aged 13–16) were motivated to make friends, yet often imitated neurotypical peers, and masked feelings of unhappiness and anxiety in social situations to prevent relationship breakdown (Tierney et al. 2016). A mixed methods investigation of the social motivation and friendship quality of adolescent autistic boys, autistic girls and their neurotypical peers (n = 46) revealed autistic boys were significantly less socially motivated than all other groups (Sedgewick et al. 2016). Interestingly, autistic girls reported similar friendship qualities to girls without autism, except in the area of conflict, where the autistic groups (both male and female) reported less conflict in their friendships than neurotypical peers. However, semi-structured interviews uncovered increased aggression within the friendships of autistic girls, suggesting difficulties identifying and potentially managing conflict within friendships (Sedgewick et al. 2016). Additional evidence of social and friendship differences between the genders in autism was reported by Baldwin and Costley (2016) who conducted a questionnaire study (n = 82) and found differences in social attitudes between the sexes. While autistic girls appeared more content in their own company in comparison to the male group, there was also evidence that autistic females find the demands and disappointments of social endeavours more of a burden on their psychological and emotional state.

Qualitative work with parents of autistic girls has also suggested possible sex-specific challenges for autistic girls including the ability to develop and maintain relationships with neurotypical girls (Cook et al. 2018; Cridland et al. 2014), masking autistic behaviours (Cook et al. 2018; Cridland et al. 2014) and coping with puberty and sexual vulnerability (Cridland et al. 2014).

The current study aims to add to this small but important body of qualitative research. This study aims to gather information from a range of perspectives, both diagnosed and self-diagnosed autistic females and parents of autistic females. We hope the broad scope of the topic guide (Appendix 1) enables reports of before, during and after diagnosis to be collected, as well as positive aspects of an autism diagnoses. By capturing the females’ first-hand accounts, we hope to improve current understanding of the female experience and to minimize the circularity of male-biased autism research.

## Methods

The current study is the first part of the third phase of the Social Relationships Study (SR Study), one of the largest population-based twin studies of cognition and behaviour across the full autism spectrum (Colvert et al. 2015). The longitudinal study has so far established the autism sample when the twins were aged 12–14 and then followed this group up at age 18 to investigate mental health and well-being in young adulthood. The third phase of the SR Study aims to investigate female autism, in terms of symptomatology and experience. However, prior to this third phase of research, a series of discussions were conducted with autistic women (both undiagnosed and diagnosed) to identify core issues and themes to be measured and avoid the circularity of relying on questionnaires and tasks derived from largely male-biased autism research.

## Participants

Participants were 18 females with a clinical diagnosis of autism (n = 16) or self-diagnosed autism (n = 2), and four mothers of autistic girls. Inclusion criteria were: (1) female gender, or parent of a female with an autism diagnosis, (2) living in the United Kingdom. Female participants in the autism group were aged between 11 and 55 years. Both clinically diagnosed and self-diagnosed individuals were invited to take part in the discussions to ensure that the groups were as inclusive as possible; thereby tackling the issue of omitting potentially misdiagnosed or undiagnosed women who have high traits of autism. Participant information can be found in Table 1.

**Table 1:** Participant information

Participant ID	Group (parent (P)/female (F))	Age range (years)	Diagnosed	Recruited from
FF01	F	20–30	N	Social media
FF02	F	40+	N	Social media
FF03	F	11–18	Y	Word of mouth
FF04	F	11–18	Y	Word of mouth
FF05	F	11–18	Y	School for autistic girls
FF06	F	11–18	Y	School for autistic girls
FF07	F	11–18	Y	School for autistic girls
FF08	F	11–18	Y	School for autistic girls
FF09	F	11–18	Y	School for autistic girls
FF10	F	20–30	Y	Social media
FF11	F	30–40	Y	BG clinic
FF12	F	40+	Y	BG clinic
FF13	F	20–30	Y	BG clinic
FF14	F	40+	Y	BG clinic
FF15	F	30–40	Y	BG clinic
FF16	F	40+	Y	BG clinic
FF17	F	40+	Y	BG clinic
FF18	F	40+	Y	Word of mouth
FP02	P	N/A	N/A	Social media
FP03	P	N/A	N/A	Social media
FP04	P	N/A	N/A	Social media
FP05	P	N/A	N/A	Word of mouth

## Procedure

Autistic females and mothers of autistic girls were recruited via four routes: adverts on social media, word of mouth, through contacts at a secondary school and through a tertiary referral autism diagnostic clinic. Participants were invited to attend group discussions at the research centre, but when this was inconvenient for the participant, in-home individual discussions and/or telephone discussions were offered. Both group and individual discussions were offered to ensure as many participants could be included as possible, and to reflect the preferences of the participants themselves.

Information sheets were provided via email and/or in person for those who met the researchers face to face. Consent was obtained either in person or via post for those who completed telephone discussions. Four group discussions were held, three of which had two participants each and were held at the research centre and one with five participants, held at a secondary school, specifically for girls with social and communication difficulties. Seven individual discussions were held, six were held at the individuals’ homes and one at the research centre. Four telephone discussions were conducted. Two researchers were present for the individual and group discussions, with the exception of telephone interviews where one researcher was present. All discussions were audio recorded and transcribed. Ethical approval was obtained from the psychiatry, nursing and midwifery (PNM) research ethics subcommittee (RESC).

## Measures

A topic guide was used for the discussions and included 15 (for the female autism group) or 16 (for the parents) questions (See Appendix 1), covering three overarching topics: diagnostic pathway, impact of autism, and resilience and coping. The questions were designed by the research team and were guided by previous research, the writings of women on the spectrum, and current hypotheses in the research field. They were designed to be used flexibly, allowing the research team to follow participants’ answers and prompt for more in-depth information as appropriate.

## Data Analysis

All discussions were audio recorded and transcribed verbatim. Two members of the research team (VM and HM) then followed Thematic Analysis guidelines (Braun and Clarke 2006) to identify themes in the data. Following initial coding, both VM and HM regularly discussed and compared codes to create themes and sub-themes and data to support the themes. The themes were checked to ensure coherence, consistency and clarity. There were no disagreements between the coders, however if disagreements were to arise a discussion was planned to be had with a third coder, until agreement was reached.

## Results

Parent discussions lasted on average 55.5 min (range = 33–93 min) and the autistic females’ individual discussions lasted on average 46.8 min (range = 12–82 min). The average duration of group discussions (average = 56.75 min) was longer than individual discussions (average 48.7 min) which is to be expected. There were no differences found in the content of the data gathered from group and individual discussions, therefore the authors collapsed the data. No adverse effects were reported by participants and no discussions were terminated before the end of questioning.

## Qualitative Analysis

From an abundance of relevant transcript data, five overarching themes comprising seventeen subthemes were identified and are presented in Table 2.

**Table 2:** Thematic analysis

Theme	Sub-theme	Example
Fitting in with the norm	Friendship motivation, conflict and maintenance	*“All my life is like I didn’t fit in, like I had friends and they weren’t like proper friends and I’d fall out with them” (FF17)*
	Living in a neurotypical world	*“She is exhausted just by the business of running an ordinary life” (FP03)*
	The concept of gender	*“Boys are more content to be themselves and it’s like this is how I am, whereas the girls really want to fit in, um, and I think that makes them unhappier” (FP04)*
	Coping strategies	*“I try to spend as much time alone as I can cos it really does like it gets me in a very calm state of mind so that when I do need to interact with people I’m willing to talk and socialise” (FF05)*
Potential obstacles for autistic women and girls	The struggle of getting a diagnosis	*“That’s the trouble with female ASD is in that time slot of whether they’re going to say yes or no to your diagnosis you could be performing or camouflaging so well that they’re not going to see that” (FF01)*
	Lack of appropriate support	*“Back in the day they didn’t really help me, they just put me down to really being a naughty child” (FF11)*
Negative aspects of autism	Co-morbid conditions	*“If you could take away that anxiety, that, then I think we could fly”(FP02)*
	Sensory sensitivities	*“The sensory issues are just, it’s the most difficult thing in the world and it’s so distressing and it really does make a difference between I think um having life quality or not for me” (FF18)*
	Meltdowns/shutdowns	*“So, shutdown I associate with myself just going like really quiet, I don’t want to interact, um, a meltdown will be like really tearful, upset, angry, distressed” (FF01)*
	Dependence/vulnerability	*“I’m prey in the world of predators” (FF07)*
	Feeling different	*“I knew that I was different, all, always knew I was different, always I knew it, in so many ways that it’s just unbelievable” (FF18)*
	Additional problems	*“Puberty, and periods, and relationships and sex and all that kind of stuff, that was incredibly difficult growing up” (FF01)*
The perspective of others	Girls can be autistic too!	*“It’s almost like, um, it would be contagious or something like that, it’s like keep my children away” (FP04)*
	Parental attitudes	*“You grieve for the child that you didn’t know you thought you had…will she ever get married; will she ever go to university…” (FP04)*
Positive aspects of autism	Benefits of autism	*“I’m starting to appreciate more and more that like the way I see the world is, can be a benefit” (FF10)*
	Accepting autism and Understanding why you’re different	*“It’s part of what makes me, me so yeah I’m happy you know it’s there, I certainly wouldn’t take it away” (FF05)*
	Strong sense of justice	*“She has this fantastic moral compass and she always wants to stick up for people” (FP02)*

Whilst some of the themes and subthemes have been identified in current literature, we deemed it important to report them in our paper to support existing findings. There were several unique subthemes revealed in our data, for example ‘living in a neurotypical world’, ‘vulnerability’, ‘feeling different’ and the theme of ‘potential obstacles for women and girls with autism’.

To ensure confidentiality, quotes are labelled with “FP” and a unique code to identify quotes from parents, or “FF” and a unique code to identify quotes from the autistic females themselves.

### Theme 1: Fitting in with the Norm

This theme encapsulates the attempts, both successful and unsuccessful, that women and girls make to attempt to fit in with their peers and society. We define “norm” as typical and/or expected behaviours.

#### Subtheme 1.1: Friendship Motivation, Conflict and Maintenance

Friendship was mentioned by the majority of the women, girls and parents as a difficulty faced by females with autism.

All my life is like I didn’t fit in, like I had friends and they weren’t like my proper friends and I’d fall out with them (FF17)

This 40-years-old woman’s quote reflected the experiences of the majority of the participants and demonstrates that although the women were able to make friends, it often felt as though they were not truly part of the group or the same as their peers. One mother made a poignant comment.

I felt at secondary school that they were kind of the left-over girls […] I did feel that they were girls that kind of drifted together because they weren’t in any other group (FP03)

Conflict within relationships was mentioned by several of the females we spoke to.

I was fed up of like getting into almost fights with people and losing my friends and alienating myself (FF17)

Maintenance of friendships was also highlighted as a problem.

I don’t think I have difficulty making friendships, it’s keeping them maybe (FF18)

The difficulties faced with friendships and fitting in with peers often led to feelings of loneliness.

Sometimes I just feel a bit sort of rejected, I do feel lonely (FF16)

It took months for me to finally get a group of friends, I remember at some points feeling depressed and totally lonely (FF04).

All the participants who discussed friendship felt as though they did want friends, and social motivation was a key theme, with all the women and girls demonstrating some desire to have friendships or social contact; however, the females commented that difficulties in social interaction made friendship building difficult.

she desperately would like to have friends and have friends invite her out and do things with her but they don’t (FP04)

I wanted to join in but I wasn’t sure how (FF02)

#### Subtheme 1.2: Living in a Neurotypical World

Difficulties with social interaction may also lead to problems in day to day life. Individuals with autism are required to live in a neurotypical world where ordinary life is often not tailored to help with problems with social interaction. Several participants commented on the difficulties they face with trying to cope in neurotypical situations, with one mother saying that, although her daughter has several positive qualities, struggling with the norm was exhausting.

She, when she’s doing her job she’s a very professional lady, but ordinary things, ordinary life exhausts her. She is exhausted just by the business of running an ordinary life (FP03)

The concept of coping with “normal” life being exhausting was mentioned by almost all the women and girls. It was highlighted that autistic females are required to adapt their thinking styles to suit the “norm” and cope with the neurotypical world.

You have a different way of viewing things and a different way of doing things which can make it harder (FF07)

The women interviewed shared a variety of different problems faced due to their autism; however, it was felt by numerous participants that if the neurotypical world had a greater understanding of autism, the problems would be almost eliminated.

If we had an understanding in society, if people respected differences and neurodiversity it wouldn’t be a problem, it really wouldn’t (FF18)

I think people need to you know, talk about it really so you know people can understand and appreciate it, you know they don’t […] people like don’t hold the person’s differences against them they can […] celebrate you know their differences (FF03)

However, one woman felt it is important to remember the unique experience everyone has.

Every girl has a completely different experience with autism (FF05)

#### Subtheme 1.3: The Concept of Gender

Within society, not just within the autism community, the participants stated that females are pressured to be more social than males, however with the added difficulties of being an individual with autism these social pressures are amplified.

There’s a lot more pressure on girls to be a certain way just in general but I think that especially affects girls on the autistic spectrum because we are more different anyway so it’s more difficult for us to be just the same as everyone else (FF13)

You have all the problems of being on the spectrum and then also all the problems of trying to be a woman on the spectrum, so trying to feel like a normal, um, woman I guess (FF10)

The difference in communication style between men and women was also discussed frequently in the discussions. It seemed that the women we interviewed felt that, in general or stereotypically, both autistic and neurotypical males and females have different styles of communication.

Like socially women just kind of like, gather round and talk and or watch things and chat and gossip, and I, I just don’t really get gossip, gossip doesn’t, I don’t know why it exists, why you do it kind of thing, but, so I always kind of I always got on with boys or men better (FF01)

Women socialise by mimicking and guys socialise by just being themselves […] if you are sort of just a little bit different you get sort of estranged from everyone (FF13)

The participants stated that as a female it was more difficult to be accepted by peers of the same gender than it was for males.

I think it’s harder, much harder as a girl because girl peers are less forgiving of other girls. The girls seem to be very tolerant of the boys with autism and almost mother them (FP04)

An alternative view was proposed by one participant, who suggested that differences are individual and not necessarily related to gender.

I guess no two people are the same are they, whether they’re male or female or both female or both male (FF15)

However, the large majority (all but one) felt there were differences between autistic males and females. It was consistently suggested that autistic males feel less pressure to mask or camouflage their symptoms, and that females were more successful at doing so.

Boys are more content to be themselves and it’s like this is how I am, whereas the girls really want to fit in, um, and I think that makes them unhappier (FP04)

I think with males, they never have this um, it’s like what I get down about is feeling like I should have to interact, and they’re more happy to say like, no I wanna do my own thing (FF01)

Two women also commented that these gender differences in masking may contribute to the different rates of females compared to males being diagnosed with autism.

I think that’s kind of the main difference that girls are just better at hiding their autism and yeah that’s probably why people […] that’s probably why people think it’s more guys who get autism because with boys it’s more obvious however girls like maybe it’s like they can just go under the radar so maybe that’s why people don’t think girls with autism exists […] or why it can take longer to get a diagnosis because again they’re just better at hiding their autism, they’re just better at masking (FF05)

It’s almost like if you put me in a room with 100 different men and some of them are autistic I would probably be able to point out which ones are autistic quite easily whereas with women it wouldn’t be that obvious (FF18)

An additional societal pressure felt by some of the women and girls we interviewed was the concept of gender itself. Gender norms are a binary cultural concept that some chose not to conform to.

gender norms, and stuff like that confuse me (FF10)

One mother stated that her daughter finds it difficult to adopt the idea of being feminine.

She chooses to wear masculine clothes because it’s so much simpler, she doesn’t then have to worry about the intricacies of make-up and things, so I think femininity is a big issue (FP03)

Two other females commented that they felt they didn’t relate to their own gender.

How kind of girls socialise, I never really related to (FF10)

I’m no good at being a girl (FF02)

Overall, the participants highlighted several gender differences and problems associated with these difficulties from the perspective of autistic females and parents.

#### Subtheme 1.4: Coping Strategies

This sub-theme identifies techniques adopted by the women to cope with their disorder. A range of specific coping strategies were mentioned by the women and girls spoken to, however three prominent mechanisms emerged.

Firstly, nearly all the participants stated that they need time alone so as not to become overwhelmed.

Both at school and at home I try to spend as much time alone as I can cause it really does like it gets me in a very calm state of mind so that when I do need to interact with people I’m willing to talk and socialise and stuff (FF05)

Secondly, the need for routine was commonly discussed.

Structure’s very important so if something like didn’t quite go to plan it would cause a bit, it would like throw me out of sync and I wouldn’t like it (FF03)

Thirdly, problems in terms of coping with “normal” everyday situations led onto the idea of masking and camouflaging autistic behaviour to fit in with a neurotypical world and disguise social interaction difficulties. All except three females reported that they camouflaged their autism symptoms.

Girls are really good at, you know, masking and hiding their autism so that it’s harder to identify an autistic girl that you know needs help with the world (FF05)

The three participants who did not report camouflaging their autism symptoms stated that they felt unable to do so as their autistic behaviour was too obvious to others.

I don’t think I have ever had to mask my autism […] I don’t think I could if I tried, I’m crazy all the way (FF07)

Camouflaged… err I’m not entirely sure that’s possible […] even if I tried it wouldn’t work or […] people would sense something quite off maybe (FF04)

Both the autistic women and girls and the mothers of autistic girls commented that neurotypical behaviour was consciously learned, for example, eye contact, in order to fit in and disguise autistic behaviour.

I didn’t want anything more than just to be normal and to fit in so I, really, really tried and I kept you know imitating and copying and making myself look and appear as normal as I could, but yeah I guess it was almost like a special interest (FF10)

The socialising bit because I was so scared, because I didn’t know what to do, so everything I had to learn by observing (FF18)

You’ve probably noticed she makes eye contact but it’s, it’s a bit clunky you know, but she’s learnt to do that (FP04)

Such masking behaviour can have implications. For example, masking behaviour during diagnostic discussions contributes to misdiagnoses and missed diagnoses.

And then you said that there’s a problem and they don’t believe you because you look fine (FF02)

The problem I’ve found is when I’m in social situations I sort of go onto auto-pilot […] and I’m kind of like polite and very British you know and so I found that in the [diagnostic] interview I was acting you know like nothing was wrong which was obviously the worst thing to do (FF13)

Although learning neurotypical behaviours may allow individuals with autism to appear “normal”, it was evident that behind the masks the women were still struggling; several commented on the immense effort it takes to maintain such behaviours.

It’s kind of like a duck on water you know it’s calm on the surface but sort of paddling really hard underneath (FF13)

Whether the females we interviewed felt they masked their autistic behaviours or not, all women and girls commented on the struggles they experienced whilst trying to fit in with a neurotypical world.

### Theme 2: Potential Obstacles for Autistic Women and Girls

This theme uncovers the barriers and difficulties faced by the women and girls. The majority of the females we spoke to had already gained a diagnosis of autism for themselves or their daughters. They discussed the difficulties they faced when trying to get a diagnosis and problems faced in terms of support after the diagnosis was received.

#### Subtheme 2.1: The Struggle of Getting a Diagnosis

Two of the women we spoke to had not yet received a diagnosis and were unsure whether they would pursue one as they had heard of others’ bad experiences. These negative experiences were reflected by the majority of the women who had been diagnosed as adults.

We headed to the nearest café and cried, cried, cried for a day; […] it was the most awful, awful experience (FP03)

It was quite a drawn-out process and quite a pain in the arse to be perfectly honest (FF13)

Despite some reports of a negative diagnostic process, many participants stated they felt relief after receiving a diagnosis.

Once I had the label that I had, I’m like yay, I’m not so crazy after all, I’m not this weird crazy person, I do fit in somewhere (FF17)

Participants reported feeling as if they understood why they had felt different, that they were relieved it was not a problem they had caused, and that they were not alone.

Fewer females are diagnosed with autism than males, and the woman and girls we spoke to suggested that this discrepancy may be due to the tools used and the design of the diagnostic process. These quotes are connected closely to the previously mentioned subtheme “masking & camouflaging”.

Girls are really good at you know, masking, and hiding their autism so that it’s harder to identify an autistic girl that you know needs help with the world (FF05)

That’s the trouble with female ASD is in that time slot of whether they’re going to say yes or no to your diagnosis you could be performing or camouflaging so well that they’re not going to see that (FF01)

The participants suggested that females are able to disguise their autism symptoms which can mean clinicians often mis-diagnosed or completely missed diagnoses.

When I actually got tested I was on autopilot and it meant that I got misdiagnosed (FF13)

#### Subtheme 2.2: Lack of Appropriate Support

Once a diagnosis was given, one woman reported that there was no after-care, or support given.

The people handling it were you know fine, were lovely, they listened and stuff but afterwards there wasn’t really any support (FF10)

Two women reported that they experienced poor support in schools, being named a “naughty child” (FF11) or a “slow learner” (FF16). One woman felt cheated by the lack of support given.

She now feels very cheated because she feels she should have had specific help, she now knows there was help she could have had that would have made her life easier (FP03)

### Theme 3: Negative Aspects of Autism

This theme explores the difficulties faced by the women and girls that are associated with having autism. Within the discussions, additional problems were discussed that, while related, did not directly involve the core diagnostic features of autism.

#### Subtheme 3.1: Co-morbid Conditions

Sixteen out of the eighteen females suffered from co-morbid conditions. Often, the women and girls had been suffering from conditions such as anxiety, OCD and depression for many years.

My depression started about 19 […] I’ve had that quite a number of years; too long (FF16)

I remember the anxiety, always the anxiety, always… being in class and thinking I know the answer but please don’t ask me (FF18)

Two women also discussed how they felt they had been misdiagnosed with a co-morbid condition instead of their autism.

I was diagnosed with depression briefly but that was obviously the Asperger’s before and so I did, I was treated for that (FF17)

So, I was really, really good at covering up my, what I thought was anxiety and social anxiety (FF10)

Often, it was these co-morbid conditions that caused the main problems in the females’ lives.

I always say I would never change anything but if I could change something it’d be the obsessive compulsive because I can see it tires her out (FP02)

I think probably the anxiety that stems from it, more than anything else […] I’ve missed out on a lot of opportunity because of like fear (FF10)

#### Subtheme 3.2: Sensory Sensitivities

Apart from co-morbid mental health conditions, sensory sensitivities were reported to play a large role in eleven of the eighteen females’ everyday lives. These sensory issues ranged from the dislike of loud noises, to powerful cross-modal effects.

When she was younger, if I had lilies in the house she’d almost go deaf…. it was like the sensory overload made something else shut down (FP04)

Although largely problematic, some sensory hyper-sensitivities were reported to be a positive experience.

I have the sensory thing as well, like music for me I feel like more intensely than other people I think; to put headphones in is almost more euphoric than a lot of people would experience (FF01)

The majority of females we spoke to, however, found sensory stimulation overwhelming and debilitating, with eight participants stating that they considered their sensory issues the most debilitating aspect of their lives.

The sensory issues are just, it’s the most difficult thing in the world and it’s so distressing and it really does make a difference between, I think, um having life quality or not for me (FF18)

#### Subtheme 3.3: Meltdowns and Shutdowns

In reaction to overwhelming emotional and sensory situations, several women and girls reported experiencing what they called “meltdowns” and “shutdowns”.

So, shutdown I associate with myself just going like really quiet, I don’t want to interact, um, a meltdown will be like really tearful, upset, angry, distressed, um it’s kind of cathartic to me sometimes (FF01)

As she got older she would, I can’t explain it any other way, close her face, literally shutdown and if, if confronted, that would lead to, you know, bad tempers, and throwing things, not meltdowns but tempers, you have to wait ‘til it came out (FP03)

Often the women/girls labelled these experiences as “overloads” (FF17).

#### Subtheme 3.4: Dependence/Vulnerability

The females often discussed feelings of vulnerability and dependence. One woman stated that she was jealous of other students in her class who did not need the help she needed. However, the most common mention of vulnerability was in terms of sexual relationships.

I was kind of naïve or gullible… towards people and they would take advantage or something like that (FF01)

You have to try and think a little bit more carefully when you’re around other people and other men, and… cause sometimes you give out the wrong body signals and people pick it up wrongly (FF16)

#### Subtheme 3.5: Feeling Different

Participants often commented on their feelings of being different to those around them from a young age.

Very different to most people, or as I like to put it, I’m prey in the world of predators (FF07)

I knew that I was different, all, always knew I was different, always I knew it, in so many ways that it’s just unbelievable (FF18)

It was often reported that the women and girls were frustrated because despite feeling that they were different in some way, they did not understand it themselves and were often misunderstood by others.

I thought I was naughty, I just felt I was very different to other children […] in how my brain processed things, I think, and how I couldn’t do what other children could do (FF11)

I knew at some stage that I was different but never really knew or understood it (FF16)

Some individuals found it frustrating and disliked feeling different from other people.

It’s frustrating for yourself if you don’t know, you know there’s something wrong with you but you don’t know what it is (FF16)

I wish I didn’t have the ASD and I wish I could just do what normal people do um, and it, I find it really hard to live with every day (FF11)

One individual reinforced that, although they might feel different, they did not feel that they were inadequate.

It’s just being different it’s not being less or anything so (FF18)

The women also noted problems they had in terms of social interaction and how frustration with not being able to understand neurotypical interaction could arise.

I certainly remember wondering, feeling like normal people have telepath-, the ability to sort of telepathy, like telling each other in their minds what they had broadcast a telepathic message saying let’s kick this friendship off by going to my house and having a party or something and I’m and it’s like I’m not telepathic, I can’t pick up any telepathic messages (FF04)

It kind of feels like you’re an outsider looking in and like there’s this world that you’re just kind of observing from the outside and when you have to get directly involved in it, it can be a bit hard sometimes (FF05)

The intricacies of social interaction can be difficult to learn and understand. Several participants commented on specific problems including not understanding humour, not knowing when to join or add to a conversation, concerns about coming across as rude, and lack of interest in “small talk”. Many women and girls commented that they preferred acting as a “wall flower” or sitting with adults when they were children, as they found social interaction easier that way. Interestingly, one woman commented that although she preferred not to socialise much, it wasn’t the socialising that troubled her, it was the lack of understanding around social interaction.

It wasn’t the socialising that scared me it was not knowing how to do it so (FF18)

Both parents and the females themselves commented on other people’s awareness, or lack of it, concerning autism.

From birth she was quite plainly different, but I hadn’t had any experience to base anything on until I started to study it myself (FP03)

Several females explicitly stated that they believed other people could notice their autism. However, the parents who commented on noticing their daughter being different said that either they had felt they were doing something wrong to cause their daughter to act differently, or they had thought their child was unique and the differences were not a problem. Two women with autism stated that their parents did not believe either the women themselves, or “in autism”.

#### Subtheme 3.6: Additional Problems

A range of other negative aspects of life with autism were reported, which did not fit into sensory issues or co-morbid conditions. Two participants commented that they have a bad memory and felt it was related to their autism.

That’s the disadvantage of my autism, I have a terrible memory (FF07)

Three women also commented that puberty and sexual relationships were difficult aspects of their lives.

Puberty, and periods, and relationships and sex and all that kind of stuff, that was incredibly difficult growing up (FF01)

### Theme 4: The Perspective of Others

This theme considers how other people, including peers and family members, understand and are impacted by autism. From speaking to mothers of autistic girls, we were able to gain information on the impact autism has on the wider family, not just the individual themselves. The mothers spoke of feeling isolated, family breakdown and narrowed social lives. Two of the autistic females we spoke to also gave insight into running their own families. One stated that being a mother is more important to them than being a autistic woman.

#### Subtheme 4.1: Girls can be Autistic Too!

The mothers, woman and girls interviewed were very passionate about a need for greater understanding of autism, and in particular autism in females. The lack of understanding of autism in the general population has caused problems for the families interviewed.

It’s almost like, um, it would be contagious or something like that, it’s like ‘keep my children away!’ (FP04)

It feels difficult like other people don’t really understand your needs, so like I’d be having a breakdown in the middle of Costa and people would be like pulling their children away and it’s like they don’t understand, they just think I’m a naughty child (FF06)

Although research is improving in the field of autism, one female expressed the need for a greater understanding of girls with autism.

I think it would be nice for people to realise that autism can affect girls (FF12)

#### Subtheme 4.2: Parental Attitudes

Mothers of autistic girls discussed their personal feelings towards having a child with autism. One mother commented on the concern they felt about their child achieving the life they anticipated for them.

You grieve for the child that you didn’t know you thought you had…will she ever get married; will she ever go to university… (FP04)

### Theme 5: Positive Aspects of Autism

This theme highlights some of the benefits of being autistic and ways females have learnt to understand their disorder. Not all aspects of having autism are negative, as reported by our participants.

#### Subtheme 5.1: Benefits of Autism

A common positive of having autism mentioned in the discussions was being able to see the world from a different and unique perspective.

I’m starting to appreciate more and more that like the way I see the world is, can be a benefit (FF10)

I’m unique in my own way that makes me feel that I am a unique part of the world (FF07)

Other benefits of having autism ranged from having long attention spans and good memory, to having an improved sense of empathy and greater creativity. Only one woman we asked was unable to think of a positive aspect of having autism.

#### Subtheme 5.2: Accepting Autism and Understanding Why You’re Different

Importantly, some participants mentioned that having autism is not a definitive feature of an individual’s life.

I know that I am different, and I don’t think it matters to anybody else if I am different because I don’t think autism is a way to define a person, it’s more the way they act, and how they feel (FF07)

#### Subtheme 5.3: Strong Sense of Justice

Interestingly, several females and parents commented on autistic females having a strong sense of justice.

It’s wonderful that she has this fantastic moral compass and she always wants to stick up for people because she’s had problems and she’s been bullied and she wants to stick up for everyone, but the way in which she wants to do it puts herself at a risk… (FP02)

The need to stand up for those who cannot stand up for themselves was a recurrent theme.

## Discussion

The current study aimed to minimise the circularity of exploring female autism within a primarily male-biased field. From speaking to the women, girls and mothers, we have gained an insight into the first-hand experiences of females who identify as being autistic and have reported a vast amount of information and a wide range of themes and subthemes.

As our topic guide included a range of open discussion points, our participants had the freedom to discuss their own experiences in depth, resulting in several holistic accounts and an abundance of information to explore. The broad variety of opinions, resulting from including females within a wide age range and stages of diagnosis, gives our project a unique quality. As we have not focused solely on one area, for example friendships or camouflaging, we are able to explore both positive and negative aspects of the female experience, and several novel themes. The experiences of the females we met overlapped considerably and the few contradictions in the data, such as not all women feeling able to mask and/or camouflage their symptoms, are discussed further within this section. Overall, the data was largely cohesive, and as autism is a heterogenous disorder, it is acceptable and expected that some disagreement in subjective experience arose.

The women and girls in this study reported adopting strategies to** mask and camouflage** their autistic behaviours. During discussions women who believed they successfully masked their symptoms reported how they might learn stock phrases in social etiquette or consciously study the “appropriate” amount of time to maintain eye contact. Similar findings have been found in previous research, for example Dean, Harwood & Kasari (2017) found that school-aged autistic girls were more likely to adopt compensatory social behaviours than their male peers, which further suggests that females are more likely to be overlooked and potentially have difficulties gaining a diagnosis.

It is unclear whether camouflaging behaviour is a protective or a harmful technique. Many women report negative consequences to this behaviour such as exhaustion and poor mental health (Bargiela et al. 2016; Lai et al. 2011). We did not include a comparison group of autistic males; therefore, it is unclear whether this strategy could be considered a specific feature of female autism (Bargiela et al. 2016). In fact, Hull et al. (2017) found both males and females report using camouflaging techniques. Interestingly, not all women in our sample felt masking and camouflaging their autism was useful or even possible; it remains unclear what drives and allows individuals to use such techniques.

Evidence from the discussions supports the premise that autistic females** struggle to initiate and maintain relationships** and resolve conflicts within friendships (Sedgewick et al. 2016; Kirkovski et al. 2013). A common misconception is that individuals with autism do not desire or seek friendships and social interaction, however, despite our participants reporting difficulties with friendship formation and maintenance, the discussions demonstrated that many autistic females are socially motivated. The women and girls we spoke to all wanted friendships, yet often reported feelings of loneliness. This finding was also reported by Sedgewick et al. (2016) who interviewed adolescent males and females and found that autistic females were as social motivated as their neurotypical counterparts, while autistic males were less socially motivated in comparison to autistic females and neurotypical males and females. Autism literature supports the idea that females are more interested in social relationships than males, perhaps indicating a somewhat distinct autism phenotype in females.

It is unclear whether the gender differences in** nuanced communication and behaviours** described by our participants are unique to an autistic population, as we did not compare our volunteers to a neurotypical sample. McVey et al. (2016) suggested that social nuances in female communication are more complex than those found within male communication. This notion was echoed by one of our participants, who claimed she found it easier to communicate with males because their communication styles were clearer, and they found relationship maintenance less challenging. Kanfiszer et al. (2017) reported similar findings, with women reporting they felt different to societal gender norms, and felt their interests were more aligned with those of male than female peers.

A general lack of understanding about female autism symptomology, including camouflaging behaviour and interest in social relationships, is suspected to lead to many women receiving a late or** delayed diagnosis** (Haney 2016; Bargiela et al. 2016; Gould and Ashton-Smith 2011; Giarelli et al. 2010). A study investigating maternal concerns also highlighted the impact of delayed diagnosis (Navot et al. 2017). Mothers reported a lack of clinician awareness of female autism and difficulties gaining a diagnostic referral despite raising concerns early. This resonates with the reports some of the women and girls in our sample gave of poor diagnostic processes, and further highlights the need for a greater understanding of female autism. Additionally, these findings possibly support the notion that diagnostic tools are biased towards male presentations of autism.

The participants in our study linked masking behaviour to delayed diagnosis and **delayed access to support** for autistic females. Tint and Weiss (2017) findings reflect this and go on to discuss various unmet needs including employment support and mental health support. Baldwin and Costley (2016) found participants who remained undiagnosed up until 18 years of age or after were much less likely to receive adequate educational support than those who received an earlier diagnosis while in education. It has also been reported by parents of autistic females that they’ve felt “at war” with schools in order to gain appropriate support for their child, and even schools not implementing individualized education plans despite the plans being put in place (Mademtzi et al. 2018).

Despite a largely negative view towards their experiences of autism, an encouraging finding is that the females identified several **positive aspects** of being autistic. Although previous research has reported the positive experience of gaining a diagnosis (Bargiela et al. 2016), more specific positive aspects such as those mentioned in our data are often underreported.

## Study Limitations

This study provides useful information towards improving understanding of the female experience of autism. However, there are some methodological factors that may limit the generalizability of these findings. Firstly, the small sample (n = 22), although typical of qualitative studies, makes it difficult to explore how race, ethnicity, background or social-economic status might affect these findings. Furthermore, as is common with qualitative studies, the small sample size means findings may not be representative of all autistic women.

Whilst the inclusion of self-diagnosed autistic females is a step in the right direction as current research literature highlights the potential for missed or misdiagnosis of females, the small number (n = 2) of participants in comparison to diagnosed females (n = 16) is not ideal. However, as this was a small-scale study, and no differences were found between the responses of self-diagnosed and diagnosed participants, the authors deemed it acceptable to include these participants in the sample. It is not known whether there is a difference between self-diagnosed autistic individuals who actively seek a diagnosis in comparison to those who do not. This would be an interesting area for future research.

Furthermore, a recruitment bias may pertain; participants in the discussions volunteered to take part. It could be argued that individuals “coping better” are more likely to volunteer to speak about their experience, and so we may not gain an accurate perspective for the full spectrum. Individuals on the autism spectrum with intellectual disability and/or minimal language are generally neglected in research and it is important to consider how their views can be gathered and how they can be brought into participatory research models (Chakrabarti 2017).

In some of the discussions, there were an equal number of researchers to participants or occasionally more researchers than participants. It is possible that this dynamic influenced the participants’ responses, however no evidence of this was found in the data.

Finally, while gender differences were a topic examined in these discussions, a comparison male autism group was not included and would be useful in future studies.

## Implications & Future Directions

The discussions highlighted important themes, including both negative and positive factors that contribute to the experiences of autistic females. We believe this research has several potential implications.

First, the negative accounts of getting an autism diagnosis emphasise the need to adapt diagnostic processes to be more inclusive for females and their families. Future guidelines could include information for clinicians to aid understanding of barriers to diagnosis for females, such as the misconception that autism is a solely male diagnosis.

Topics for future research have been identified: one suggestion is to explore whether camouflaging behaviours are adopted by individuals with a diagnosis other than autism or no diagnoses at all, and whether there is a gender disparity in camouflaging behaviours within these populations.

Finally, for autistic females, having the opportunity to share their experiences and perspectives contribute towards viewing autism with a gender balanced lens, rather than the current male focus. It’s hoped that a greater understanding of female autism will allow autistic individuals to receive better recognition and understanding, and thus have a more positive experience.

## Conclusion

By capturing qualitative accounts of the female experiences of autism, we hope to contribute to a greater understanding of the obstacles and challenges faced by women and girls at various stages of having an autism diagnosis. We have also reported several positive aspects of autism, which are often underreported in the literature. We hope that the information gathered, and the small glimpse into the lives of autistic females, can influence future research and clinical practice, and has given autistic females the opportunity to share their too-often ignored voices.

## Electronic Supplementary Material

Below is the link to the electronic supplementary material.

Supplementary material 1 (DOC 279 KB)

## References

[CR1] American, Psychiatric Association (2013). *Diagnostic and statistical manual of mental disorders* (*DSM-5®*).

[CR2] Attwood T (2006). The complete guide to Asperger’s syndrome.

[CR3] Baldwin S, Costley D (2016). The experiences and needs of female adults with high-functioning autism spectrum disorder. Autism.

[CR4] Bargiela S, Steward R, Mandy W (2016). The experiences of late-diagnosed women with autism spectrum conditions: An investigation of the female autism phenotype. Journal of Autism and Developmental Disorders.

[CR5] Braun V, Clarke V (2006). Using thematic analysis in psychology. Qualitative Research in Psychology.

[CR6] Cage E, Troxell-Whitman Z (2019). Understanding the reasons, contexts, and costs of camouflaging for autistic adults. Journal of Autism and Developmental Disorders.

[CR7] Chakrabarti B (2017). Commentary: Critical considerations for studying low-functioning autism. Journal of Child Psychology and Psychiatry.

[CR8] Colvert E, Tick B, McEwen F, Stewart C, Curran SR, Woodhouse E, Ronald A (2015). Heritability of autism spectrum disorder in a UK population-based twin sample. JAMA Psychiatry.

[CR9] Cook A, Ogden J, Winstone N (2018). Friendship motivations, challenges and the role of masking for girls with autism in contrasting school settings. European Journal of Special Needs Education.

[CR10] Cridland EK, Jones SC, Caputi P, Magee CA (2014). Being a girl in a boys’ world: Investigating the experiences of girls with autism spectrum disorders during adolescence. Journal of Autism and Developmental Disorders.

[CR11] Dean M, Harwood R, Kasari C (2017). The art of camouflage: Gender differences in the social behaviors of girls and boys with autism spectrum disorder. Autism.

[CR12] Dworzynski K, Ronald A, Bolton P, Happé F (2012). How different are girls and boys above and below the diagnostic threshold for autism spectrum disorders?. Journal of the American Academy of Child & Adolescent Psychiatry.

[CR13] Giarelli E, Wiggins LD, Rice CE, Levy SE, Kirby RS, Pinto-Martin J, Mandell D (2010). Sex differences in the evaluation and diagnosis of autism spectrum disorders among children. Disability and Health Journal.

[CR14] Gould J, Ashton-Smith J (2011). Missed diagnosis or misdiagnosis? Girls and women on the autism spectrum. Good Autism Practice (GAP).

[CR15] Haney JL (2016). Autism, females, and the DSM-5: Gender bias in autism diagnosis. Social Work in Mental Health.

[CR16] Hull L, Petrides KV, Allison C, Smith P, Baron-Cohen S, Lai MC, Mandy W (2017). “Putting on my best normal”: Social camouflaging in adults with autism spectrum conditions. Journal of Autism and Developmental Disorders.

[CR17] Kanfiszer L, Davies F, Collins S (2017). ‘I was just so different’: The experiences of women diagnosed with an autism spectrum disorder in adulthood in relation to gender and social relationships. Autism.

[CR18] Kim YS, Leventhal BL, Koh YJ, Fombonne E, Laska E, Lim EC, Song DH (2011). Prevalence of autism spectrum disorders in a total population sample. American Journal of Psychiatry.

[CR19] Kirkovski M, Enticott PG, Fitzgerald PB (2013). A review of the role of female gender in autism spectrum disorders. Journal of Autism and Developmental Disorders.

[CR20] Kopp S, Gillberg C (1992). Girls with social deficits and learning problems: Autism, atypical asperger syndrome or a variant of these conditions. European Child & Adolescent Psychiatry.

[CR21] Lai MC, Lombardo MV, Auyeung B, Chakrabarti B, Baron-Cohen S (2015). Sex/gender differences and autism: setting the scene for future research. Journal of the American Academy of Child & Adolescent Psychiatry.

[CR22] Lai MC, Lombardo MV, Pasco G, Ruigrok AN, Wheelwright SJ, Sadek SA, MRC AIMS Consortium (2011). A behavioral comparison of male and female adults with high functioning autism spectrum conditions. PLoS ONE.

[CR25] Loomes R, Hull L, Mandy WPL (2017). What is the male-to-female ratio in autism spectrum disorder? A systematic review and meta-analysis. Journal of the American Academy of Child & Adolescent Psychiatry.

[CR26] Mademtzi M, Singh P, Shic F, Koenig K (2018). Challenges of females with autism: A parental perspective. Journal of Autism and Developmental Disorders.

[CR27] McVey AJ, Dolan BK, Willar KS, Pleiss S, Karst JS, Casnar CL, Van Hecke AV (2016). A replication and extension of the PEERS® for young adults social skills intervention: Examining effects on social skills and social anxiety in young adults with autism spectrum disorder. Journal of Autism and Developmental Disorders.

[CR28] Navot N, Jorgenson AG, Webb SJ (2017). Maternal experience raising girls with autism spectrum disorder: A qualitative study. Child: Care, Health and Development.

[CR29] Pellicano E, Dinsmore A, Charman T (2014). What should autism research focus upon? Community views and priorities from the United Kingdom. Autism.

[CR30] Sedgewick F, Hill V, Yates R, Pickering L, Pellicano E (2016). Gender differences in the social motivation and friendship experiences of autistic and non-autistic adolescents. Journal of Autism and Developmental Disorders.

[CR31] Tierney S, Burns J, Kilbey E (2016). Looking behind the mask: Social coping strategies of girls on the autistic spectrum. Research in Autism Spectrum Disorders.

[CR32] Tint A, Weiss JA (2017). A qualitative study of the service experiences of women with autism spectrum disorder. Autism.

