# Supplementary material for: A Qualitative Exploration of the Female Experience of Autism Spectrum Disorder (ASD)
Source: J Autism Dev Disord. 2019 Feb 21;49(6):2389–402. doi: 10.1007/s10803-019-03906-4 (PMC6546643; doi:10.1007/s10803-019-03906-4)
Supplement: Supplementary file 1 — Supplementary material 1 (DOC 279 KB) [file 10803_2019_3906_MOESM1_ESM.doc]

**
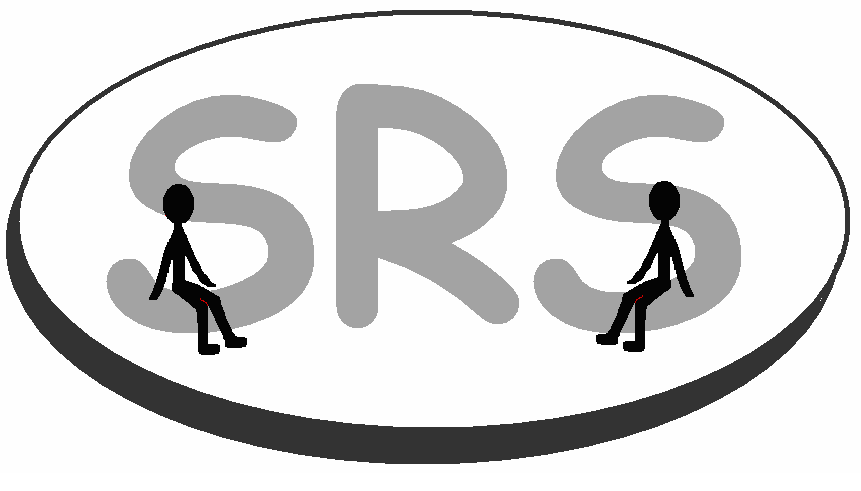
**

**Focus Group Topic Guide**

Social Relationships Study Phase Three – Gender Differences in ASD

| Date of focus group |  |
| --- | --- |
| Number of people present |  |
| Written informed consent obtained from all? |  |
| Consent to audio record focus group? |  |
| Initials of people present and professional background |  |
|  |
|  |
|  |
|  |
|  |
|  |
|  |
|  |

**General Introductions**

Thank you for agreeing to come along to this focus group. We are very interested to hear your valuable opinions. [Moderator introduces self and the notetaker.]

The aim of this meeting is to generate discussion about female ASD, about the things that are important to you, things you might find challenging and things you do to help in day to day life.

*Review the following:*

- Who we are and what we’re trying to do
- What will be done with this information

**Explanation of the process**

Ask the group if anyone has participated in a focus group before.

*About focus groups*

- We learn from you (positive and negative).
- Not trying to achieve consensus, we’re gathering information.

*Logistics*

- Focus group will last about one hour.
- Feel free to move around.
- Where are the toilets? Exit?
- Help yourself to refreshments.

*Ground rules*

- Confirm that participants can withdraw from the study at any time during or up to 2 weeks after the focus group.
- During the group all refer to each other using first names only.
- The information given is completely confidential, and we will not associate names with anything said in the focus group.
- Respect for other’s opinions.
- Only one person to speak at a time.
- There are no right or wrong answers.
- You do not have to speak in any particular order.
- Avoid disclosure of confidential clinical information.
- Focus group will be audio recorded so that we can make sure to capture the thoughts, opinions, and ideas we hear from the group. No names will be attached to the focus group and the recordings will be destroyed as soon as they are transcribed.
- If there are any concerns regarding issues discussed, to let the moderator know.

**Turn on MP3 Recorder**

1. Ask the group if there are any questions before we get started, and address those questions.
2. Topics/Themes

*Discussion begins, make sure to give people time to think before answering the questions and don’t move too quickly. Use probes to make sure that all issues are addressed, but move on when you feel you are starting to hear repetitive information.*

For those Diagnosed with ASD:

**Diagnostic pathway**:

When and how did you become aware of your ASD?

How did you seek help?

What has been your experience of the process of being diagnosed?

How aware were others of your difficulties, family, school?

Do you have any other diagnoses, if so what are these? When were they made?

For all groups:

**Impact of ASD:**

What is it like to be a woman with ASD?

What is difficult about having ASD?

What is positive about having ASD?

What are the most important implications?

Do you think there is anything different about female and male ASD?

**Resilience and Coping:**

Is there anything that has helped you to cope?

What strategies do you use to help you in day to day life?

Have you ever masked or camouflaged your ASD? How?

Is there a cost to the strategies you have used?

Parent and Sibling focus group:

**Diagnostic pathway**:

When and how did you become aware that your relative had ASD?

How did they seek help, did you help with this process?

What has been your experience of the process of them being diagnosed?

Do they have any other diagnoses, if so what are these? When did they get them?

**Impact of ASD:**

What do you think it is like to be a woman with ASD?

What is it like to have a relative with ASD?

What is difficult about them having ASD?

What is positive about them having ASD?

What are the most important implications?

Do you think there is anything different about female and male ASD?

**Resilience and Coping:**

Is there anything that has helped them to cope?

How much help have you been in terms of resilience and coping and how much have they worked out for themselves?

What strategies do they use to help in day to day life?

Have they ever masked or camouflaged their ASD? How?

Is there a cost to the strategies they have used?

**Concluding question**

Of all the things we’ve discussed today, what would you say are the most important issues you would like to express?

**Conclusion**

- Thank you for participating. This has been a very successful discussion.
- Your opinions will be a valuable asset to the study.
- We hope you have found the discussion interesting.
